# Elevated levels of body mass index and waist circumference, but not high variability, are associated with an increased risk of atrial fibrillation

**DOI:** 10.1186/s12916-022-02413-1

**Published:** 2022-06-29

**Authors:** Maoxiang Zhao, Lulu Song, Qianqian Zhao, Yating Chen, Bin Li, Zhonghui Xie, Zihao Fu, Nan Zhang, Xiaowei Cheng, Xiaoqian Li, Miao Wang, Shouling Wu, Hao Xue, Yang Li

**Affiliations:** 1 Department of Cardiology, The First Medical Center, Chinese People’s Liberation Army Hospital, Medical School of Chinese People’s Liberation Army, Beijing, China; 2 Department of Maternal and Child Health, School of Public Health, Tongji Medical College, Huazhong University of Science and Technology, Wuhan, Hubei China; 3 Department of Cardiology, Fujian Medical University, Fuzhou, China; 4 School of Medicine, Nankai University, Tianjin, China; 5 Department of Cardiology, Kailuan Hospital, Tangshan, China; 6 Department of Cardiology, the Sixth Medical Center, Chinese People’s Liberation Army Hospital, Medical School of Chinese People’s Liberation Army, Beijing, China

**Keywords:** Atrial fibrillation, Body mass index, Obesity, Variability, Waist circumference

## Abstract

**Background:**

Although obesity has been associated with risk of atrial fibrillation (AF), the associations of variability of obesity measures with AF risk are uncertain, and longitudinal studies among Chinese population are still lacking. We aimed to evaluate the impacts of obesity and variability of body mass index (BMI) and waist circumference (WC) on the risk of atrial fibrillation (AF) in a large Chinese cohort study.

**Methods:**

A total of 44,135 participants of the Kailuan Study who were free of cancer and cardiovascular disease and underwent three consecutive surveys from 2006 to 2010 were followed for incident AF until 2020. Average BMI and WC over time and variability were calculated. Cox proportional hazards regression models were used to assess hazard ratios (HRs) and 95% confidence intervals (CIs) for the associations of obesity and variability in BMI and WC with AF risk.

**Results:**

During a mean follow-up of 9.68 years, there were 410 cases of incident AF. In multivariable-adjusted models, compared with normal BMI/WC, individuals with general obesity and abdominal obesity had increased risk of AF, with corresponding HRs of 1.73 (95% CI: 1.31–2.30) and 1.38 (95% CI: 1.11–1.60), respectively. The short-term elevation in AF risk persisted for the obese even after adjustment for updated biologic intermediaries and weight. Variability in BMI and WC were not associated with the risk of AF. The restricted cubic spline models indicated significant linear relationships between levels of WC and BMI and risk of AF.

**Conclusions:**

Elevated levels of BMI and WC were associated with an increased risk of AF, whereas variability in BMI and WC were not. Therefore, achieving optimal levels of BMI and WC could be valuable in AF prevention.

**Supplementary Information:**

The online version contains supplementary material available at 10.1186/s12916-022-02413-1.

## Background

Atrial fibrillation (AF), the most commonly sustained arrhythmia in the general population, is a severe/growing public health issue afflicting millions of people worldwide [1]. It is well-established that overweight or obesity is strongly associated with an increased risk of incident AF [2–9]. However, the consensus regarding the patterns of association between body mass index (BMI) and AF risk is still lacking. Some studies have reported a linear relationship between BMI and AF risk [6, 10], whereas a J-shaped relationship was observed in other studies [2, 7]. In addition, most previous studies were based on BMI levels and did not take into account BMI changes that occur during follow-up.

In recent years, BMI variability has been proposed as a potential risk factor for adverse outcomes [11–15]. Accumulating evidence has shown that BMI variability was positively associated with risk of myocardial infarction, heart failure, stroke, and all-cause mortality [12, 13]. However, the data available for the association between BMI variability and AF were limited and the findings were inconsistent [2, 14, 16]. For instance, a large cohort study from Norway reported that higher BMI variability was associated with an increased risk of AF [2]. However, in a Korean study, BMI variability was not associated with new-onset AF in overweight/obese individuals [14]. In addition, BMI was used as a measure of obesity in previous studies, which is not a good estimator of body fat distribution compared with waist circumference (WC) [17]. Little is known about the impacts of WC level and its variability on AF.

To address these gaps in our knowledge, we investigated the associations of both levels of BMI and WC and their variabilities with the risk of AF in a large community-based Chinese cohort.

## Methods

### Study population

We used the data from the Kailuan Study, an ongoing prospective cohort study conducted at 11 hospital affiliated with the Kailuan Group from June 2006 to October 2007 in Tangshan, northern China. All the participants in the Kailuan Study are employees and retirees of the Kailuan Group, which is a coal mining company in Tangshan. A detailed study design has been published elsewhere [18, 19]. In brief, this cohort consists of 101,510 adult participants enrolled at 1st survey between 2006 and 2007. All the participants underwent questionnaire assessments, clinical examinations, and laboratory tests biennially.

**Fig. 1:**
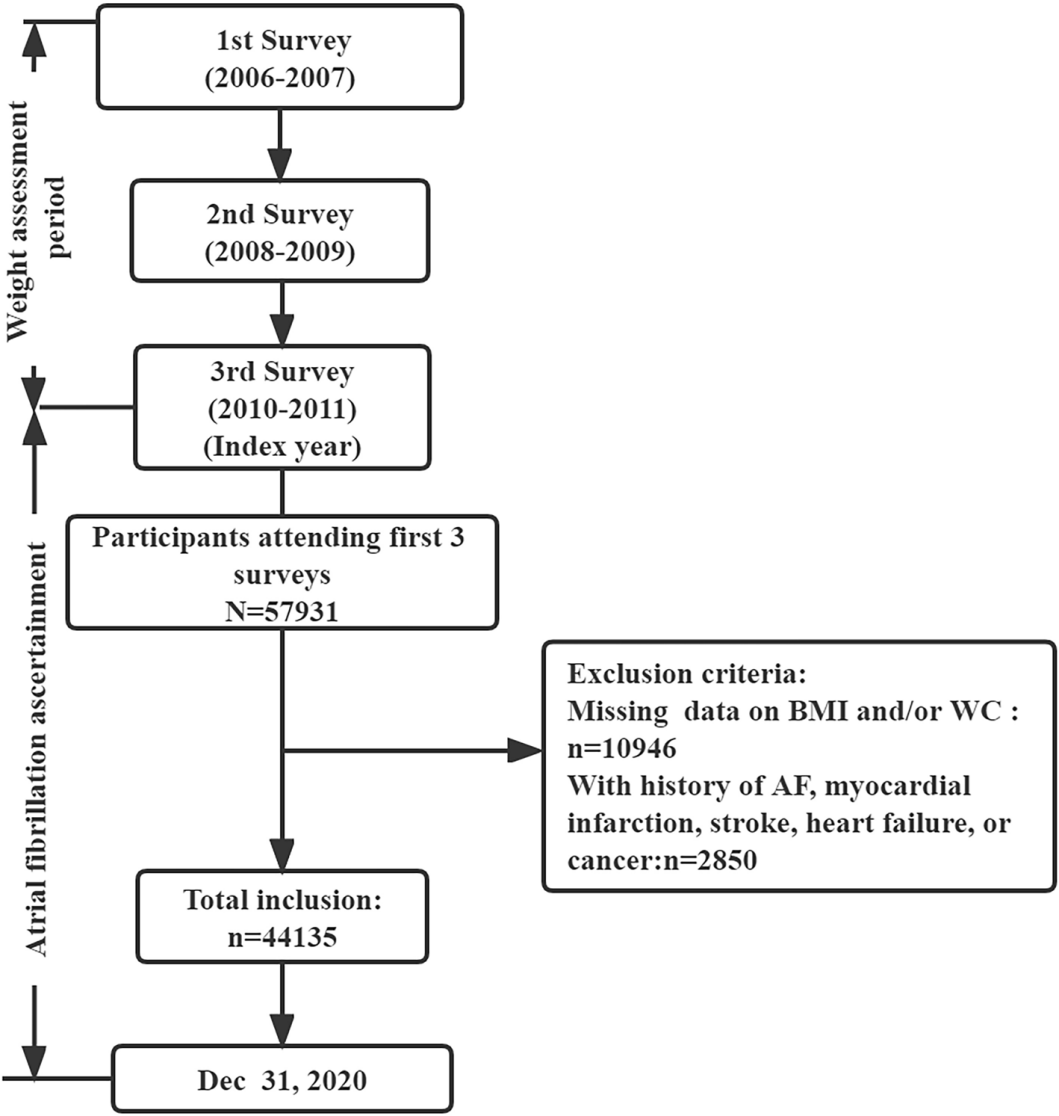
Flow chart of the current study

In the current study, 57,931 participants underwent three consecutive surveys at 2006–2007, 2008–2009, and 2010–2011 (index year). Of these remaining participants, we excluded those with missing data on BMI or WC (*n*=10,946) and with history of AF, myocardial infarction, stroke, heart failure, or cancer prior to 2010–2011 (*n*=2850). Ultimately, the study population included 44135 participants (Fig. 1). The study protocol was approved by the ethics committee of the Kailuan General Hospital. All the participants gave written informed consent prior to study inclusion.

### Assessment of BMI and WC

Height and weight were measured while participants wore light clothing without shoes. BMI was calculated as weight divided by the squared height (kg/m^2^). WC was defined as the smallest perimeter located between the last rib and the iliac crest, rounded to the nearest inch, and measured by the trained physicians. Obesity was classified by BMI and WC criteria, respectively. BMI was categorized according to the recommendations of the Working Group on Obesity in China [20]: Underweight (BMI < 18.5 kg/m^2^), normal weight (BMI 18.5–23.9 kg/m^2^), overweight (BMI 24–27.9 kg/m^2^), and obese (BMI ≥ 28 kg/m^2^). Abdominal obesity was defined as WC ≥ 90 cm for men and ≥ 80 cm for women, as recommended by the International Diabetes Federation (IDF) for Chinese adults [21].

Average BMI and WC were calculated using the following formula:

$$\mathrm{Average}\ \mathrm{BMI}=\left[\left({\mathrm{BMI}}_{06}\times {\mathrm{Time}}_{1-2}\right)+\left({\mathrm{BMI}}_{08}\times {\mathrm{Time}}_{2-3}\right)\right]/{\mathrm{Time}}_{1-3}$$

$$\mathrm{Average}\ \mathrm{WC}=\left[\left({\mathrm{WC}}_{06}\times {\mathrm{Time}}_{1-2}\right)+\left({\mathrm{WC}}_{08}\times {\mathrm{Time}}_{2-3}\right)\right]/{\mathrm{Time}}_{1-3}$$

$${\mathrm{Time}}_{1-2}=\mathrm{time}\ \mathrm{from}\ 1\mathrm{st}\ \mathrm{survey}\ \left(2006-2007\right)\ \mathrm{to}\ 2\mathrm{nd}\ \mathrm{survey}\ \left(2008-2010\right);$$

$${\mathrm{Time}}_{2-3}=\mathrm{time}\ \mathrm{from}\ 2\mathrm{nd}\ \mathrm{survey}\ \left(2008-2009\right)\ \mathrm{to}\ 3\mathrm{rd}\ \mathrm{survey}\ \left(2010-2011\right);$$

$${\mathrm{Time}}_{1-3}=\mathrm{time}\ \mathrm{from}\ 1\mathrm{st}\ \mathrm{survey}\ \left(2006-2007\right)\ \mathrm{to}\ 3\mathrm{rd}\ \mathrm{survey}\ \left(2010-2011\right);$$

$${\mathrm{BMI}}_{06}=\mathrm{value}\ \mathrm{of}\ \mathrm{BMI}\ \mathrm{at}\ 1\mathrm{st}\ \mathrm{survey}\ \left(2006-2007\right)$$

$${\mathrm{BMI}}_{08}=\mathrm{value}\ \mathrm{of}\ \mathrm{BMI}\ \mathrm{at}\ 2\mathrm{nd}\ \mathrm{survey}\ \left(2008-2010\right)$$

$${\mathrm{WC}}_{06}=\mathrm{value}\ \mathrm{of}\ \mathrm{WC}\ \mathrm{at}\ 1\mathrm{st}\ \mathrm{survey}\ \left(2006-2007\right)$$

$${\mathrm{WC}}_{08}=\mathrm{value}\ \mathrm{of}\ \mathrm{WC}\ \mathrm{at}\ 2\mathrm{nd}\ \mathrm{survey}\ \left(2008-2010\right)$$

### Assessment of variability in BMI and WC

Variability in BMI and WC were assessed across three measures (in years 2006–2007, 2008–2009, and 2010–2011). Four indices of variability were used: standard deviation (SD), coefficient of variation (CV), average real variability (ARV), and variability independent of the mean (VIM). ARV is the average of the absolute differences between consecutive values and was calculated using the following formula: $ARV=\frac{1}{n-1}{\sum}_{i-1}^{n-1}\left|{value}_{i+1}-\left.{value}_i\right|\right.$, where N denotes the number of measurements of BMI and WC. VIM was calculated as *M*^*beta*^ × *SD*/*mean*^*beta*^, where M was the average of mean BMI or WC among the study participants, beta was derived from fitting curves, and SD=k × mean^x^ [22, 23].

### Covariates

A face-to-face interview was conducted at each survey by trained staffs via standardized questionnaires to collect information on age, sex (male or female), medical history (e.g., cardiovascular disease, diabetes and hypertension), smoking status (never, former, or current smoker), alcohol drinking status (never, former, or current alcohol drinker), physical activity (never, 1–2 times/week, or ≥ 3 times/week). Current smokers were defined as those who smoked at least one cigarette per day for more than six months. Current alcohol drinkers were defined as those drank at least once per month for more than six months. Physical activity was evaluated according to the frequency of physical activity (≥ 30 min/time) during leisure time and was divided into never, 1–2 times/week, and ≥ 3 times/week. Laboratory tests, including triglycerides (TG), total cholesterol (TC), low-density lipoprotein cholesterol (LDL-C), high-density lipoprotein cholesterol (HDL-C), and fasting blood glucose (FBG) were performed by analyzer (Hitachi Automation Analyzer, Tokyo, Japan). Blood pressure (BP) was measured in a sitting upright position after at least 5 min of rest. Diabetes was defined as a FBG level ≥ 7.0 mmol/L, or current use of hypoglycemic medication, or self-reported physician-diagnosed diabetes [24]. Hypertension was defined as systolic BP (SBP)/diastolic BP (DBP) ≥ 140/90 mmHg, or use of antihypertensive medication, or self-reported physician-diagnosed hypertension [25].

### Ascertainment of outcome

The primary outcome of the current study was the first occurrence of AF. Potential AF cases were ascertained from the Municipal Social Insurance Institution that covered all the participants, the Hospital Discharge Register from 11 affiliated hospitals, and questionnaire survey (biennially since 2006). Potential AF cases were identified following the International Classification of Diseases, Tenth Revision (ICD-10) codes. For suspected AF cases, a panel of three physicians reviewed the medical records and confirmed the AF diagnosis. During biennial interview, all participants underwent a 10-s 12-lead ECG examination. AF was diagnosed based on the following ECG criteria: (1) irregular R-R intervals; (2) absence of repeating P waves; and (3) irregular atrial activity, according to the European Society of Cardiology guidelines [26]. Mortality information was obtained from provincial vital statistics offices and updated annually. Participants were followed up from 3^rd^ survey until the date of death, occurrence of AF, or 31 December 2020.

### Statistical analysis

Categorical variables were expressed as numbers and percentages, and continuous variables were expressed as mean ± standard deviation (SD). Comparisons of continuous variables were conducted using the analysis of variance, whereas categorical variables were compared using the Chi-square test. The incidence rate of AF was calculated by dividing the number of incident cases by the total follow-up duration (person-years).

Cox proportional hazards regression models were used to calculate the hazard ratios (HRs) and 95% confidence intervals (CIs) for the associations of levels of BMI and WC and their variabilities with the risk of AF. Levels of BMI and WC were assessed both as continuous and categorical variables, and variability of BMI and WC were assessed as a continuous variable. All the Cox proportional hazards models were adjusted for age, sex, physical activity, smoking status, alcohol drinking status, LDL-C, heart rate, hypertension, and diabetes. To account for changes in levels of BMI and WC over time and assess the short-term effects of BMI and WC on risk of incident AF, we conducted time-dependent Cox regression models where levels of BMI and WC were updated at each follow-up and the most recent measurements were used to estimate risk during the follow-up time period. Restricted cubic spline models with three knots at the 25th, 50th, and 75th percentiles were used to explore the patterns of associations between levels of BMI and WC and their variabilities and risk of AF.

Several sensitivity analyses were performed to assess the robustness of our findings: (1) excluding participants with incident AF or death occurring within the first year of follow-up; (2) excluding the participants developing CVD during follow-up; (3) defining variability in BMI and WC using four measures (in years 2006–2007, 2008–2009, 2010–2011, and 2012–2013); (4) stratifying the analyses by diabetes status (yes or no); and (5) using Fine-Gray models instead, accounting for the competing risk of death.

All statistical analyses were conducted using SAS 9.4 (SAS Institute; Cray, NC). Tests were 2-sided, and a *P* value < 0.05 was considered statistically significant. The proportional hazards assumptions were appropriate in main models.

## Results

### Baseline characteristics

Of the 44,135 participants, 78.4% were men and the mean±SD age was 48.76±11.73 years. The baseline characteristics of participants according to BMI and WC categories are presented in Table 1 and Additional file 1: Table S1. Compared with those with normal BMI, participants in higher BMI categories were more likely to be physically active, had higher proportions of diabetes and hypertension, and had higher SBP, DBP, and LDL-C. Participants in higher WC categories were more likely to be older and women, were less likely to be current smokers and current alcohol drinkers, had higher proportions of diabetes and hypertension, and had higher SBP, DBP, and LDL-C than those with normal WC. The correlation between BMI and WC is shown in Additional file 1: Fig. S1. BMI was positively correlated with WC (*r* = 0.747).

**Table 1:** Characteristics of participants according to BMI categories (*n*=44,135)

	Underweight (BMI<18kg/m^2^)	Normal weight (18.5≤BMI<24kg/m^2^)	Overweight (24≤BMI<28kg/m^2^)	Obesity (BMI≥28kg/m^2^)
No. of participants	523	17107	19187	7318
Age, year	46.53±15.35	48.04±12.12	49.47±11.19	48.65±11.80
Male	348 (66.54)	12811 (74.89)	15662 (81.63)	5757 (78.67)
Physical activity ≥3 times/week	56 (11.09)	2112 (12.68)	2583 (13.86)	1018 (14.28)
Current smoker	148 (29.19)	5894 (35.32)	6530 (34.98)	2308 (32.30)
Current alcohol drinker	149 (29.45)	6262 (37.52)	7359 (39.41)	2689 (37.59)
Diabetes	14 (2.68)	1094 (6.40)	2252 (11.74)	1269 (17.34)
Hypertension	120 (22.94)	5676 (33.18)	9497 (49.50)	4524 (61.82)
SBP, mmHg	119.53±17.61	126.30±18.30	132.82±18.62	137.41±18.86
DBP, mmHg	77.55±9.82	81.77±10.22	85.57±10.39	88.59±10.95
LDL, mmol/L	2.33±0.78	2.52±0.81	2.65±0.83	2.69±0.84
Heart Rate, beats/min	75.14±11.07	73.48±10.34	73.36±10.07	74.07±10.36

Values are presented as the mean ± SD or *n* (%)

*Abbreviations*: *BMI* Body mass index, *DBP* Diastolic blood pressure, *LDL* Low-density lipoprotein, *SBP* Systolic blood pressure

### Average BMI and WC and AF risk

**Fig. 2:**
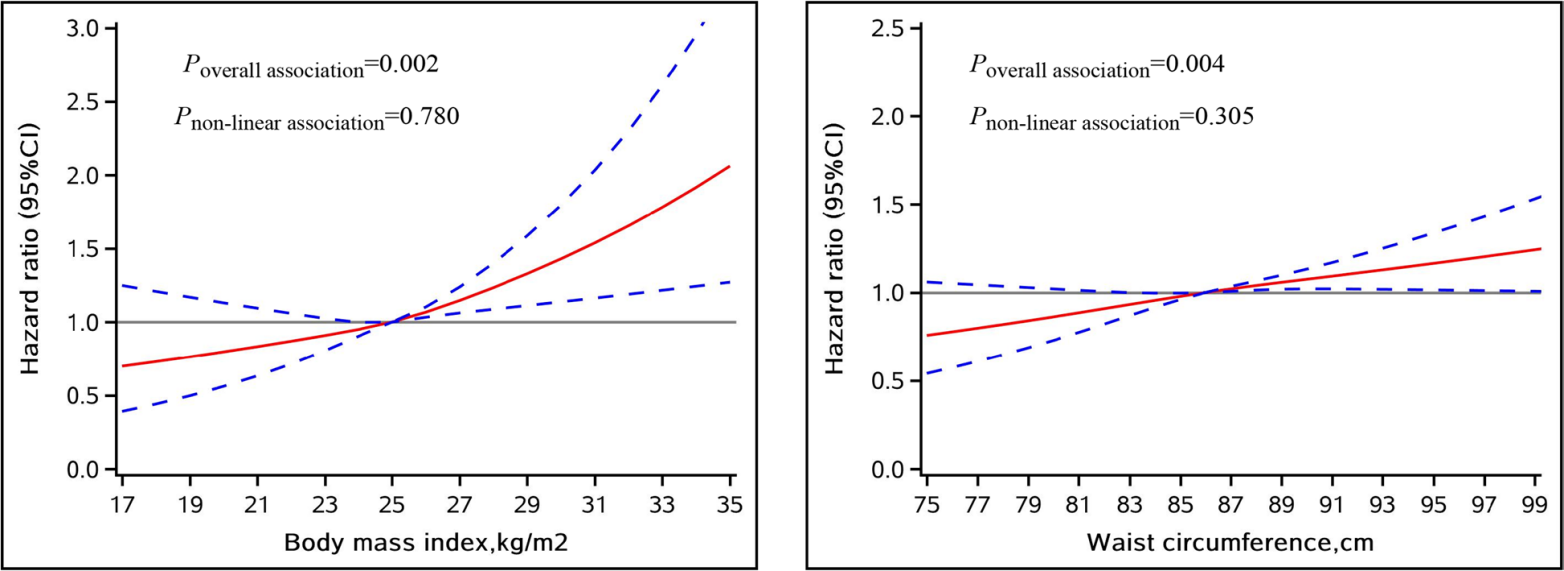
Associations of average BMI and WC with risk of atrial fibrillation using restricted cubic spline regression models. Point estimates (solid line) and 95% confidence intervals (dashed lines) were obtained by restricted cubic spline models with knots at the 25th, 50th, and 75th percentiles. All models were adjusted for age, sex, physical activity, smoking status, alcohol drinking status, low-density lipoprotein cholesterol, heart rate, hypertension, and diabetes. BMI, body mass index; CI, confidence interval; HR, hazard ratio; WC, waist circumference

During a mean follow-up of 9.68 years (427110 person-years), 410 participants developed AF. The overall incidence rate for AF was 0.96 per 1000 person-years. The associations of average BMI and WC with AF risk are shown in Table 2. When evaluating BMI and WC as categorical variables, the multivariable-adjusted HRs for general and abdominal obesity when compared with normal BMI/WC were 1.73 (95% CI: 1.31–2.30) and 1.38 (95% CI: 1.11–1.60), respectively. For the continuous BMI and WC, each 1-unit increase in BMI and WC was associated with 6% (95% CI: 1.03–1.10) and 2% (95% CI: 1.01–1.03) higher AF risk, respectively. In time-dependent Cox regression models introducing WC, BMI, and confounders as time-varying covariates, the associations of BMI and WC with AF risk became stronger compared with the original analysis (Table 2). The cubic spline models showed linear positive relationships between BMI and WC and risk of incident AF (*P*_non-linear association_ = 0.780 and *P*_non-linear association_ =0.305, respectively) (Fig. 2).

**Table 2:** Associations of average BMI and WC with risk of atrial fibrillation

	Event/Total	Incidence rate (per 1000 persons-years)	Multivariable-adjusted HR (95% CI)^a^	HR (95% CI) in model using time-dependent variables^b^
Average BMI categories				
Underweight	3/523	0.60	0.77 (0.25, 2.44)	1.18 (0.55, 2.53)
Normal weight	132/17,107	0.80	1.00 (reference)	1.00 (reference)
Overweight	177/19,187	0.95	1.15 (0.90, 1.46)	1.28 (1.02, 1.62)
Obesity	98/7318	1.39	1.73 (1.31, 2.30)	1.89 (1.45, 2.46)
Continuous (per kg/m^2^)			1.06 (1.03, 1.10)	1.07 (1.04, 1.10)
Average WC categories				
Abdominal obesity (−)	182/24222	0.76	1.00 (reference)	1.00 (reference)
Abdominal obesity (+)	228/19913	1.21	1.38 (1.11, 1.60)	1.46 (1.17, 1.78)
Continuous (per cm)			1.02 (1.01, 1.03)	1.02 (1.01, 1.03)

*Abbreviations*: *BMI* Body mass index, *CI* Confidence interval, *HR* Hazard ratio, *WC* Waist circumference

^a^Models were adjusted for age, sex, physical activity, smoking status, alcohol drinking status, low-density lipoprotein cholesterol, heart rate, hypertension, and diabetes

^b^The models with BMI/WC, physical activity, smoking status, alcohol drinking status, low-density lipoprotein cholesterol, heart rate, hypertension, and diabetes as time-dependent variables and age and sex as time-fixed variables

### Variability in BMI and WC and AF risk

Table 3 presents associations of variability in BMI and WC with the risk of AF. There were no significant associations between variability in BMI and WC and risk of AF. Spline curves between variability in BMI and WC and the hazard ratio of new-onset AF are displayed in Additional file 1: Fig. S2 and S3. The four indices of variability, namely VIM, SD, CV, and ASV, observed consistent associations of variability in BMI and WC with risk of AF.

**Table 3:** Associations of variability in BMI and WC with risk of atrial fibrillation

Variability measures	Multivariable adjusted HR (95% CI)	
	BMI	WC
VIM	0.96 (0.86, 1.07)	1.02 (0.99, 1.05)
CV	0.99 (0.96, 1.02)	1.02 (0.99, 1.04)
ARV	0.97 (0.89, 1.05)	1.02 (0.99, 1.03)
SD	0.98 (0.89, 1.09)	1.02 (0.99, 1.04)

*Abbreviations*: *ARV* Average real variability; *BMI* Body mass index, *CV* Coefficient of variation, *CI* Confidence interval, *HR* Hazard ratio, *SD* Standard deviation, *VIM* Variability independent of the mean, *WC* Waist circumference

All models were adjusted for age, sex, physical activity, smoking status, alcohol drinking status, low-density lipoprotein cholesterol, heart rate, hypertension, and diabetes

### Sensitivity analyses

A series of sensitivity analyses were performed to examine the associations of levels of BMI and WC and their variabilities with AF risk. The results remain consistent after excluding participants with incident AF or death occurring within the first year of follow-up (Additional file 1: Tables S2 and S3), or excluding the participants developing CVD during follow-up (Additional file 1: Tables S4 and S5), or defining variability in BMI and WC using four measures (Additional file 1: Table S6). In analyses stratified by diabetes status, the associations of BMI and WC with AF risk were more evident in individuals without diabetes (Additional file 1: Table S7). In the competing risk analyses, the results from Fine-Gray model were similar to the main results (Additional file 1: Tables S8 and S9).

## Discussion

In this large community-based prospective study, we investigate the relative importance of levels of BMI and WC and their variabilities in predicting future risk of AF. We found that higher average levels of BMI and WC were linearly associated with an increased risk of AF. However, there were no significant associations between variability in BMI and WC and risk of AF.

Although a growing body of literature has shown that obesity was strongly associated with development of AF [2–9], most previous studies were based on single time point assessment of BMI and mainly focused on Caucasian. For example, a meta-analysis of 29 prospective studies suggested that general obesity and higher BMI increased the risk of AF [3]. In a prospective cohort study of UK, baseline BMI was associated with an increased risk of AF [5]. Our study extends the present knowledge as we explore the cumulative effect of BMI on AF risk in Chinese population. We found that general obesity was associated with an increased risk of AF, when compared with normal BMI. Each 1 kg/m^2^ increase in BMI was associated with a 6% increased risk of AF. This is in line with previous studies [5, 27–29], where one unit BMI increment has been described to independently increase the risk of new-onset AF of 3–8%. Moreover, utilizing updated measures of BMI, a significant short-term increase in AF risk with elevated BMI was found in our study.

Although obesity is usually quantified by the calculation of BMI, WC is a more specific measurement for body fat distribution [17]. Generally, Asians tend to have lower BMI and a higher proportion of body fat than Caucasians [30]. WC has previously been shown to provide additional predictive information on coronary artery disease and all-cause mortality [31, 32], but the relationship between WC and AF risk has received little attention, especially in China. In the current study, WC was positively associated with the risk of incident AF. In line with previous studies [6, 33], our study suggested that associations with AF appear to be stronger for BMI compared with WC. To our knowledge, this is one of the first studies examining the short-term influence of central obesity on the development of AF with updated measures of WC. The results are consistent with studies based on a single measure of WC [4, 6, 34].

In addition to BMI and WC levels, in recent year researchers have proposed BMI or WC variability as a novel predictor for adverse outcomes [11, 12, 14, 15, 35]. However, the evidence for the association between BMI variability and new-onset AF was lacking. In the HUNT study, Feng et al. [2] found that increased BMI variability was associated with an increased AF risk. A Korean nationwide cohort study reported that BMI variability was associated with the new-onset AF in underweight or normal-weight individuals, but not in overweight/obese individuals [14]. To date, the relationship between WC variability and the risk of AF has not been reported. In our study, we found no significant associations of variability in BMI and WC with the risk of AF. Several hypotheses may explain this inconsistency. First, although the adverse effect of BMI or WC variability maybe its association with elevations in cardiometabolic traits and chronic disease [11, 12, 36–38], it is still possible that variability in BMI or WC is merely a reflection of comorbid conditions that act as important confounders in determining the clinical outcomes rather than a truly causal relationship. Second, higher variability in BMI and WC may reflect changes of lifestyle factors. Therefore, adding together favorable and adverse changes may result in errors in assessment of the true impact. Although we have adjusted for several lifestyle factors including physical activity, smoking status, and alcohol drinking status at baseline, these and other lifestyle factors changes over time could lead to the degree of variability in BMI and WC. Hence, whether variability in BMI and WC are potential predictors for the incident AF warrants further investigations.

Our findings have important implications for the public health policy regarding prevention of AF in China. AF is a refractory disease associated with significant morbidity and mortality and the burden of AF has exponentially risen in recent decades, especially in Asia-Pacific region [39]. The current study demonstrated that elevated levels of BMI and WC, but not their variabilities, were associated with AF development.

Clinical and public health interventions should be aimed at maintaining optimal levels of BMI and WC and controlling general and central obesity, which are simple way to prevent AF development. Further studies are needed to explore whether higher variability in BMI and WC are associated with the risk of incident AF.

This study has several strengths. The main strength is the long follow-up of a large sample of participants with the availability of detailed information on biological variables. Second, data of weight and height were collected via direct measurement rather than self-reported. Third, this is the first cohort study to explore the relationships between both levels of BMI and WC and their variabilities and incident AF in Chinese population. We also acknowledge several limitations in our study. First, due to the inherent nature of the observational study, a causal relationship between obesity and AF development could not be determined. Second, classification of types of AF was not available in our study, as we did not have that information in the registry. Third, AF cases were mainly ascertained through hospital discharge codes, and therefore asymptomatic or undiagnosed AF cases would have been missed, which might lead to an underestimated of new-onset AF. Nevertheless, the incidence rate of AF in the current study was comparable with that reported in other studies of Chinese population [40, 41]. Fourth, our study measured variability of BMI and WC based on only three time points; it is therefore possible that we may have underestimated variability. The sensitivity analysis that defining variability in BMI and WC using four measures also revealed similar results. Finally, given that our study sample was limited to Chinese occupational population, this could limit generalizability of our findings.

## Conclusions

In this large community-based cohort study of Chinese population, elevated levels of BMI and WC, but not their variabilities, were associated with an increased risk of AF. Taken together, our findings highlight the importance of achieving optimal levels of BMI and WC to effectively prevent the development of AF.

## Supplementary Information

**Additional file 1: Table S1.** Characteristics of participants according to WC categories. **Table S2.** Associations of average BMI and WC with risk of atrial fibrillation: Sensitivity analysis excluding outcome events within the first year of follow-up. **Table S3.** Associations of variability in BMI and WC with risk of atrial fibrillation: Sensitivity analysis excluding outcome events within the first year of follow-up. **Table S4.** Associations of average BMI and WC with risk of atrial fibrillation: Sensitivity analysis excluding the participants developing CVD during follow-up. **Table S5.** Associations of variability in BMI and WC with risk of atrial fibrillation: Sensitivity analysis excluding the participants developing CVD during follow-up. **Table S6.** Associations of variability in BMI and WC with risk of atrial fibrillation: Sensitivity analysis defining variability in BMI and WC using four measures. **Table S7.** Associations of average BMI and WC with risk of atrial fibrillation: Sensitivity analysis stratifying the analyses by diabetes status. **Table S8.** Associations of average BMI and WC with risk of atrial fibrillation: Competing risk analysis. **Table S9.** Associations of variability in BMI and WC with risk of atrial fibrillation: Competing risk analysis. **Fig. S1.** Correlation between body mass index and waist circumference. **Fig. S2.** Associations of BMI variability with risk of atrial fibrillation using restricted cubic spline regression models. **Fig. S3.** Associations of WC variability with risk of atrial fibrillation using restricted cubic spline regression models.

## Data Availability

The data that support the findings of this study are available from [third party name] but restrictions apply to the availability of these data, which were used under license for the current study, and so are not publicly available. Data are however available from the corresponding author upon reasonable request and with permission of the corresponding author

## Abbreviations

AF: Atrial fibrillation
ARV: Average real variability
BMI: Body mass index
CV: Coefficient of variability
DBP: Diastolic blood pressure
HDL-C: High-density lipoprotein cholesterol
LDL-C: Low-density lipoprotein cholesterol
SBP: Systolic blood pressure
SD: Standard deviation
TG: Triglyceride
VIM: Variability independent of the mean
WC: Waist circumference

## References

[1] Brundel B, Ai X, Hills MT, Kuipers MF, Lip G, de Groot N (2022). Atrial fibrillation. Nat Rev Dis Primers.

[2] Feng T, Vegard M, Strand LB (2019). Weight and weight change and risk of atrial fibrillation: the HUNT study. Eur Heart J.

[3] Aune D, Sen A, Schlesinger S, Norat T, Janszky I, Romundstad P (2017). Body mass index, abdominal fatness, fat mass and the risk of atrial fibrillation: a systematic review and dose-response meta-analysis of prospective studies. Eur J Epidemiol.

[4] Baek YS, Yang PS, Kim TH, Uhm JS, Park J, Pak HN, et al. Associations of Abdominal Obesity and New-Onset Atrial Fibrillation in the General Population. J Am Heart Assoc. 2017;6(6). 10.1161/JAHA.116.004705. 10.1161/JAHA.116.004705 PMC5669144 28588091

[5] Neefs J, Boekholdt SM, Khaw KT, Luben R, Pfister R, Wareham NJ (2019). Body mass index and body fat distribution and new-onset atrial fibrillation: Substudy of the European Prospective Investigation into Cancer and Nutrition in Norfolk (EPIC-Norfolk) study. Nutr Metab Cardiovasc Dis.

[6] Poorthuis M, Sherliker P, de Borst GJ, Carter JL, Lam K, Jones NR (2021). Joint Associations Between Body Mass Index and Waist Circumference With Atrial Fibrillation in Men and Women. J Am Heart Assoc.

[7] Singleton MJ, German CA, Carnethon M, Soliman EZ, Bertoni AG, Yeboah J (2021). Race, Body Mass Index, and the Risk of Atrial Fibrillation: The Multi-Ethnic Study of Atherosclerosis. J Am Heart Assoc.

[8] Zia I, Johnson L, Memarian E, Borné Y, Engström G (2021). Anthropometric measures and the risk of developing atrial fibrillation: a Swedish Cohort Study. BMC Cardiovasc Disord.

[9] Tedrow UB, Conen D, Ridker PM (2010). The long- and short-term impact of elevated body mass index on the risk of new atrial fibrillation the WHS (women's health study). J Am Coll Cardiol.

[10] Tedrow UB, Conen D, Ridker PM, Cook NR, Koplan BA, Manson JE (2010). The long- and short-term impact of elevated body mass index on the risk of new atrial fibrillation the WHS (women's health study). J Am Coll Cardiol..

[11] Kaze AD, Santhanam P, Erqou S, Ahima RS, Bertoni AG, Echouffo-Tcheugui JB (2022). Body Weight Variability and Risk of Cardiovascular Outcomes and Death in the Context of Weight Loss Intervention Among Patients With Type 2 Diabetes. JAMA Netw Open.

[12] Kim MK, Han K, Park YM, Kwon HS, Kang G, Yoon KH (2018). Associations of Variability in Blood Pressure, Glucose and Cholesterol Concentrations, and Body Mass Index With Mortality and Cardiovascular Outcomes in the General Population. Circulation.

[13] Kwon S, Lee SR, Choi EK, Lee SH, Han KD, Lee SY (2019). Visit-to-visit variability of metabolic parameters and risk of heart failure: A nationwide population-based study. Int J Cardiol.

[14] Lim YM, Yang PS, Jang E, Yu HT, Kim TH, Uhm JS (2019). Body Mass Index Variability and Long-term Risk of New-Onset Atrial Fibrillation in the General Population: A Korean Nationwide Cohort Study. Mayo Clin Proc.

[15] Kaze AD, Erqou S, Santhanam P, Bertoni AG, Ahima RS, Fonarow GC (2022). Variability of adiposity indices and incident heart failure among adults with type 2 diabetes. Cardiovasc Diabetol.

[16] Lee SR, Choi EK, Han KD, Lee SH, Oh S (2020). Effect of the variability of blood pressure, glucose level, total cholesterol level, and body mass index on the risk of atrial fibrillation in a healthy population. Heart Rhythm.

[17] Snijder MB, van Dam RM, Visser M, Seidell JC (2006). What aspects of body fat are particularly hazardous and how do we measure them. Int J Epidemiol.

[18] Zhao M, Song L, Sun L (2021). Associations of Type 2 Diabetes Onset Age With Cardiovascular Disease and Mortality: The Kailuan Study. Diabetes Care.

[19] Zhang Q, Zhou Y, Gao X (2013). Ideal cardiovascular health metrics and the risks of ischemic and intracerebral hemorrhagic stroke. Stroke.

[20] Zhou BF (2002). Predictive values of body mass index and waist circumference for risk factors of certain related diseases in Chinese adults--study on optimal cut-off points of body mass index and waist circumference in Chinese adults. Biomed Environ Sci.

[21] Alberti KG, Zimmet P, Shaw J (2005). The metabolic syndrome--a new worldwide definition. Lancet.

[22] Juhanoja EP, Niiranen TJ, Johansson JK (2017). Outcome-Driven Thresholds for Increased Home Blood Pressure Variability. Hypertension.

[23] Fukuda K, Kai H, Kamouchi M (2015). Day-by-Day Blood Pressure Variability and Functional Outcome After Acute Ischemic Stroke: Fukuoka Stroke Registry. Stroke.

[24] Standards of medical care in diabetes--2010. Diabetes Care. 2010;33(Suppl 1):S11–61. 10.2337/dc10-S011. 10.2337/dc10-S011 PMC2797382 20042772

[25] Chobanian AV, Bakris GL, Black HR, Cushman WC, Green LA, Izzo JL (2003). Seventh report of the Joint National Committee on Prevention, Detection, Evaluation, and Treatment of High Blood Pressure. Hypertension.

[26] Camm AJ, Kirchhof P, Lip GY, Schotten U, Savelieva I, Ernst S (2010). Guidelines for the management of atrial fibrillation: the Task Force for the Management of Atrial Fibrillation of the European Society of Cardiology (ESC). Eur Heart J.

[27] Wang TJ, Parise H, Levy D (2004). Obesity and the risk of new-onset atrial fibrillation. JAMA.

[28] Frost L, Hune LJ, Vestergaard P (2005). Overweight and obesity as risk factors for atrial fibrillation or flutter: the Danish Diet, Cancer, and Health Study. Am J Med.

[29] Dublin S, French B, Glazer NL (2006). Risk of new-onset atrial fibrillation in relation to body mass index. Arch Intern Med.

[30] Deurenberg P, Deurenberg-Yap M, Guricci S (2002). Asians are different from Caucasians and from each other in their body mass index/body fat per cent relationship. Obes Rev.

[31] Lofgren I, Herron K, Zern T, West K, Patalay M, Shachter NS (2004). Waist circumference is a better predictor than body mass index of coronary heart disease risk in overweight premenopausal women. J Nutr.

[32] Staiano AE, Reeder BA, Elliott S, Joffres MR, Pahwa P, Kirkland SA (2012). Body mass index versus waist circumference as predictors of mortality in Canadian adults. Int J Obes (Lond).

[33] Zhang X, Zhang S, Li Y (2009). Association of obesity and atrial fibrillation among middle-aged and elderly Chinese. Int J Obes (Lond).

[34] Hamada R, Lee JS, Mori K, Watanabe E, Muto S (2018). Influence of abdominal obesity and habitual behaviors on incident atrial fibrillation in Japanese. J Cardiol.

[35] Wu M, Shu Y, Wang L, Song L, Chen S, Liu Y (2021). Visit-to-visit variability in the measurements of metabolic syndrome components and the risk of all-cause mortality, cardiovascular disease, and arterial stiffness. Nutr Metab Cardiovasc Dis.

[36] Waring ME, Eaton CB, Lasater TM, Lapane KL (2010). Incident diabetes in relation to weight patterns during middle age. Am J Epidemiol.

[37] Neiberg RH, Wing RR, Bray GA (2012). Patterns of weight change associated with long-term weight change and cardiovascular disease risk factors in the Look AHEAD Study. Obesity (Silver Spring).

[38] Zhang H, Tamakoshi K, Yatsuya H, Murata C, Wada K, Otsuka R (2005). Long-term body weight fluctuation is associated with metabolic syndrome independent of current body mass index among Japanese men. Circ J.

[39] Wong CX, Brown A, Tse HF (2017). Epidemiology of Atrial Fibrillation: The Australian and Asia-Pacific Perspective. Heart Lung Circ.

[40] Guo Y, Tian Y, Wang H, Si Q, Wang Y, Lip G (2015). Prevalence, incidence, and lifetime risk of atrial fibrillation in China: new insights into the global burden of atrial fibrillation. Chest.

[41] Chien KL, Su TC, Hsu HC, Chang WT, Chen PC, Chen MF (2010). Atrial fibrillation prevalence, incidence and risk of stroke and all-cause death among Chinese. Int J Cardiol.

