# Supplementary material for: Elevated levels of body mass index and waist circumference, but not high variability, are associated with an increased risk of atrial fibrillation
Source: BMC Med. 2022 Jun 29;20:215. doi: 10.1186/s12916-022-02413-1 (PMC9241273; doi:10.1186/s12916-022-02413-1)
Supplement: Supplementary file 1 — Additional file 1: Table S1. Characteristics of participants according to WC categories. Table S2. Associations of average BMI and WC with risk of atrial fibrillation: Sensitivity analysis excluding outcome events within the first year of follow-up. Table S3. Associations of variability in BMI and WC with risk of atrial fibrillation: Sensitivity analysis excluding outcome events within the first year of follow-up. Table S4. Associations of average BMI and WC with risk of atrial fibrillation: Sensitivity analysis excluding the participants developing CVD during follow-up. Table S5. Associations of variability in BMI and WC with risk of atrial fibrillation: Sensitivity analysis excluding the participants developing CVD during follow-up. Table S6. Associations of variability in BMI and WC with risk of atrial fibrillation: Sensitivity analysis defining variability in BMI and WC using four measures. Table S7. Associations of average BMI and WC with risk of atrial fibrillation: Sensitivity analysis stratifying the analyses by diabetes status. Table S8. Associations of average BMI and WC with risk of atrial fibrillation: Competing risk analysis. Table S9. Associations of variability in BMI and WC with risk of atrial fibrillation: Competing risk analysis. Fig. S1. Correlation between body mass index and waist circumference. Fig. S2. Associations of BMI variability with risk of atrial fibrillation using restricted cubic spline regression models. Fig. S3. Associations of WC variability with risk of atrial fibrillation using restricted cubic spline regression models. [file 12916_2022_2413_MOESM1_ESM.docx]

**Supplemental Material**

**Supplemental Table S1.** Characteristics of participants according to WC categories **(n=44135)**.

**Supplemental Table S2.** Associations of average BMI and WC with risk of atrial fibrillation**: Sensitivity analysis excluding outcome events within the first year of follow-up (n=43886).**

**Supplemental Table S3.** Associations of variability in BMI and WC with risk of atrial fibrillation: **Sensitivity analysis excluding outcome events within the first year of follow-up (n=43886).**

**Supplemental Table S4.** Associations of average BMI and WC with risk of atrial fibrillation**: Sensitivity analysis excluding the participants developing CVD during follow-up (n=41229).**

**Supplemental Table S5.** Associations of variability in BMI and WC with risk of atrial fibrillation: **Sensitivity analysis excluding the participants developing CVD during follow-up (n=41229).**

**Supplemental Table S6.** Associations of variability in BMI and WC with risk of atrial fibrillation: **Sensitivity analysis** **defining variability in BMI and WC using four measures (n=41229).**

**Supplemental Table S7.** Associations of average BMI and WC with risk of atrial fibrillation: **Sensitivity analysis** **stratifying the analyses by diabetes status (n=44135).**

**Supplemental Table S8.** Associations of average BMI and WC with risk of atrial fibrillation: **Competing risk analysis (n=44135).**

**Supplemental Table S9.** Associations of variability in BMI and WC with risk of atrial fibrillation: **Competing risk analysis (n=44135).**

**Supplemental Fig. S1.** Correlation between body mass index and waist circumference.

**Supplemental Fig. S2.** Associations of BMI variability with risk of atrial fibrillation using restricted cubic spline regression models.

**Supplemental Fig. S3.** Associations of WC variability with risk of atrial fibrillation using restricted cubic spline regression models.

**Supplemental Table S1. Characteristics of participants according to WC categories (n=44135).**

|  | Abdominal obesity (-)  (WC < 90 cm for men  WC < 80 cm for women) | Abdominal obesity (+)  (WC ≥ 90 cm for men  WC≥ 80 cm for women) |
| --- | --- | --- |
| No. of participants | 24805 | 19330 |
| Age, year | 47.38±11.92 | 50.49±11.26 |
| Male | 21141 (85.23) | 13437 (69.51) |
| Physical activity ≥3 times/week | 3352 (13.75) | 2417 (13.04) |
| Current smoker | 9470 (38.79) | 5410 (29.10) |
| Current alcohol drinker | 10249 (41.97) | 6210 (33.38) |
| Diabetes | 1742 (7.02) | 2887 (14.94) |
| Hypertension | 9417 (37.96) | 10400 (53.80) |
| SBP, mmHg | 127.98±18.29 | 134.65±19.27 |
| DBP, mmHg | 83.06±10.38 | 86.36±10.89 |
| LDL, mmol/L | 2.57±0.76 | 2.64±0.90 |
| Heart Rate, beats/min | 73.46±10.41 | 73.65±10.02 |

Values are presented as the mean ± SD, or n (%).

Abbreviations: DBP, diastolic blood pressure; LDL, low-density lipoprotein; SBP, systolic blood pressure; WC, waist circumference.

**Supplemental Table S2.** Associations of average BMI and WC with risk of atrial fibrillation**: Sensitivity analysis excluding outcome events within the first year of follow-up (n=43886).**

|  | Multivariable adjusted  HR (95% CI) |
| --- | --- |
|  |  |
| Average BMI categories | |
| Underweight | 0.69 (0.17, 2.82) |
| Normal weight | 1.00 (reference) |
| Overweight | 1.14 (0.87, 1.49) |
| Obese | 1.70 (1.24, 2.33) |
| Continuous (per kg/m^2^) | 1.08 (1.02, 1.12) |
| Average WC categories | |
| Abdominal obesity (-) | 1.00 (reference) |
| Abdominal obesity (+) | 1.30 (1.02, 1.65) |
| Continuous (per cm) | 1.03 (1.01, 1.04) |

Abbreviations: BMI, body mass index; CI, confidence interval; HR, hazard ratio; WC, [waist circumference](C:/Users/zmx/Documents/%E8%BD%AF%E4%BB%B6/Dict/8.9.9.0/resultui/html/index.html#/javascript:;).

All models were adjusted for age, sex, physical activity, smoking status, alcohol drinking status, low-density lipoprotein cholesterol, heart rate, hypertension, and diabetes.

**Supplemental Table S3.** Associations of variability in body mass index and waist circumference with risk of atrial fibrillation: **Sensitivity analysis excluding outcome events within the first year of follow-up (n=43886).**

| Variability measures | Multivariable adjusted HR (95% CI) | |
| --- | --- | --- |
|  | BMI | WC |
| VIM | 0.93 (0.82, 1.05) | 1.02 (0.99, 1.05) |
| CV | 0.98 (0.99, 1.94) | 1.02 (0.99, 1.04) |
| ARV | 0.97 (0.88, 1.06) | 1.02 (0.99, 1.03) |
| SD | 0.97 (0.87, 1.09) | 1.02 (0.99, 1.05) |

Abbreviations: ARV, average real variability; BMI, body mass index; CV, coefficient of variation; CI, confidence interval; HR, hazard ratio; SD, standard deviation; VIM, variability independent of the mean; WC, [waist circumference](C:/Users/zmx/Documents/%E8%BD%AF%E4%BB%B6/Dict/8.9.9.0/resultui/html/index.html#/javascript:;).

All models were adjusted for age, sex, physical activity, smoking status, alcohol drinking status, low-density lipoprotein cholesterol, heart rate, hypertension, and diabetes.

**Supplemental Table S4.** Associations of average BMI and WC with risk of atrial fibrillation**: Sensitivity analysis excluding the participants developing CVD during follow-up(n=41229).**

|  | Multivariable adjusted HR (95% CI) |
| --- | --- |
|  |  |
| Average BMI categories | |
| Underweight | 0.84 (0.27, 2.65) |
| Normal weight | 1.00 (reference) |
| Overweight | 1.16 (0.91, 1.48) |
| Obese | 1.59 (1.18, 2.13) |
| Continuous (per kg/m^2^) | 1.08 (1.04, 1.11) |
| Average WC categories | |
| Abdominal obesity (-) | 1.00 (reference) |
| Abdominal obesity (+) | 1.31 (1.06, 1.64) |
| Continuous (per cm) | 1.03 (1.01, 1.04) |

Abbreviations: BMI, body mass index; CI, confidence interval; HR, hazard ratio; WC, [waist circumference](C:/Users/zmx/Documents/%E8%BD%AF%E4%BB%B6/Dict/8.9.9.0/resultui/html/index.html#/javascript:;).

All models were adjusted for age, sex, physical activity, smoking status, alcohol drinking status, low-density lipoprotein cholesterol, heart rate, hypertension, and diabetes.

**Supplemental Table S5.** Associations of variability in body mass index and waist circumference with risk of atrial fibrillation: **Sensitivity analysis excluding the participants developing CVD during follow-up (n=41229).**

| Variability measures | Multivariable adjusted HR (95% CI) | |
| --- | --- | --- |
|  | BMI | WC |
| VIM | 0.98 (0.88, 1.10) | 1.03 (0.99, 1.05) |
| CV | 0.99 (0.97, 1.02) | 1.02 (0.99, 1.05) |
| ARV | 0.98 (0.90, 1.06) | 1.02 (0.99, 1.04) |
| SD | 1.01 (0.91, 1.12) | 1.02 (0.98, 1.05) |

Abbreviations: ARV, average real variability; BMI, body mass index; CV, coefficient of variation; CI, confidence interval; HR, hazard ratio; SD, standard deviation; VIM, variability independent of the mean; WC, [waist circumference](C:/Users/zmx/Documents/%E8%BD%AF%E4%BB%B6/Dict/8.9.9.0/resultui/html/index.html#/javascript:;).

All models were adjusted for age, sex, physical activity, smoking status, alcohol drinking status, low-density lipoprotein cholesterol, heart rate, hypertension, and diabetes.

**Supplemental Table S6.** Associations of variability in BMI and WC with risk of atrial fibrillation: **Sensitivity analysis** **defining variability in BMI and WC using four measures (n=34930).**

| Variability measures | Multivariable adjusted HR (95% CI) | |
| --- | --- | --- |
|  | BMI | WC |
| VIM | 1.04 (0.91, 1.18) | 1.03 (0.99, 1.07) |
| CV | 1.01 (0.98, 1.04) | 1.03 (0.99, 1.06) |
| ARV | 1.02 (0.92, 1.14) | 1.03 (1.00, 1.05) |
| SD | 1.07 (0.95, 1.20) | 1.04 (0.99, 1.07) |

Abbreviations: ARV, average real variability; BMI, body mass index; CV, coefficient of variation; CI, confidence interval; HR, hazard ratio; SD, standard deviation; VIM, variability independent of the mean; WC, [waist circumference](file:///E:\文章\肥胖与房颤\写稿\改稿\新建文件夹\4次变异性.docx#/javascript:;).

All models were adjusted for age, sex, physical activity, smoking status, alcohol drinking status, low-density lipoprotein cholesterol, heart rate, hypertension, and diabetes.

**Supplemental Table S7.** Associations of average BMI and WC with risk of atrial fibrillation: **Sensitivity analysis** **stratifying the analyses by diabetes status (n=44135).**

|  | Multivariable adjusted HR (95% CI) | |
| --- | --- | --- |
|  | Non-diabetes (N=39506) | Diabetes (N=4629) |
| Average BMI categories | |  |
| Underweight | 0.92 (0.29, 2.92) | - |
| Normal weight | 1.00 (reference) | 1.00 (reference) |
| Overweight | 1.14 (0.88, 1.48) | 1.38 (0.69, 2.75) |
| Obese | 1.61 (1.17, 2.22) | 1.72 (0.82, 3.65) |
| Average WC categories | |  |
| Abdominal obesity (-) | 1.00 (reference) | 1.00 (reference) |
| Abdominal obesity (+) | 1.31 (1.03, 1.66) | 1.34 (0.77, 2.35) |

Abbreviations: BMI, body mass index; CI, confidence interval; HR, hazard ratio; WC, [waist circumference](C:/Users/zmx/Documents/%E8%BD%AF%E4%BB%B6/Dict/8.9.9.0/resultui/html/index.html#/javascript:;).

All models were adjusted for age, sex, physical activity, smoking status, alcohol drinking status, low-density lipoprotein cholesterol, heart rate, and hypertension.

**Supplemental Table S8.** Associations of variability in body mass index and waist circumference with risk of atrial fibrillation: **Competing risk analysis.**

|  | Multivariable adjusted HR (95% CI) |
| --- | --- |
| Average BMI categories |  |
| Underweight | 0.85 (0.27, 2.67) |
| Normal weight | 1.00 (reference) |
| Overweight | 1.17 (0.92, 1.49) |
| Obese | 1.62 (1.21, 2.18) |
| Average WC categories |  |
| Abdominal obesity (-) | 1.00 (reference) |
| Abdominal obesity (+) | 1.31 (1.05, 1.64) |

Abbreviations: BMI, body mass index; CI, confidence interval; HR, hazard ratio; WC, [waist circumference](C:/Users/zmx/Documents/%E8%BD%AF%E4%BB%B6/Dict/8.9.9.0/resultui/html/index.html#/javascript:;).

All models were adjusted for age, sex, physical activity, smoking status, alcohol drinking status, low-density lipoprotein cholesterol, heart rate, hypertension, and diabetes.

**Supplemental Table S9.** Associations of variability in body mass index and waist circumference with risk of atrial fibrillation: **Competing risk analysis.**

| Variability measures | Multivariable adjusted HR (95% CI) | |
| --- | --- | --- |
|  | BMI | WC |
| VIM | 0.94 (0.83, 1.07) | 1.02 (0.99, 1.04) |
| CV | 0.99 (0.95, 1.02) | 1.02 (0.99, 1.04) |
| ARV | 0.96 (0.86, 1.06) | 1.01 (0.99, 1.03) |
| SD | 0.97 (0.87, 1.09) | 1.02 (0.99, 1.04) |

Abbreviations: ARV, average real variability; BMI, body mass index; CV, coefficient of variation; CI, confidence interval; HR, hazard ratio; SD, standard deviation; VIM, variability independent of the mean; WC, [waist circumference](C:/Users/zmx/Documents/%E8%BD%AF%E4%BB%B6/Dict/8.9.9.0/resultui/html/index.html#/javascript:;).

All models were adjusted for age, sex, physical activity, smoking status, alcohol drinking status, low-density lipoprotein cholesterol, heart rate, hypertension, and diabetes.

**
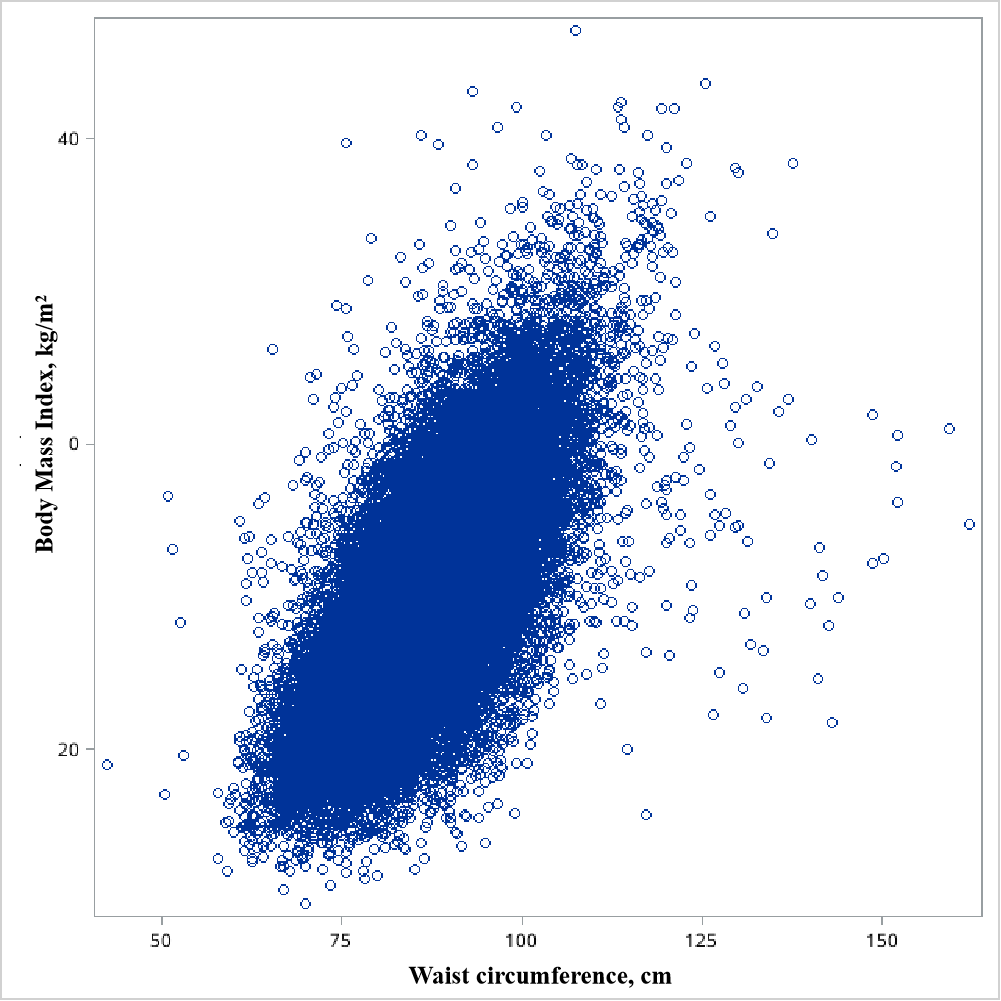
**

**Supplemental Fig. S1.** Correlation between body mass index and waist circumference.


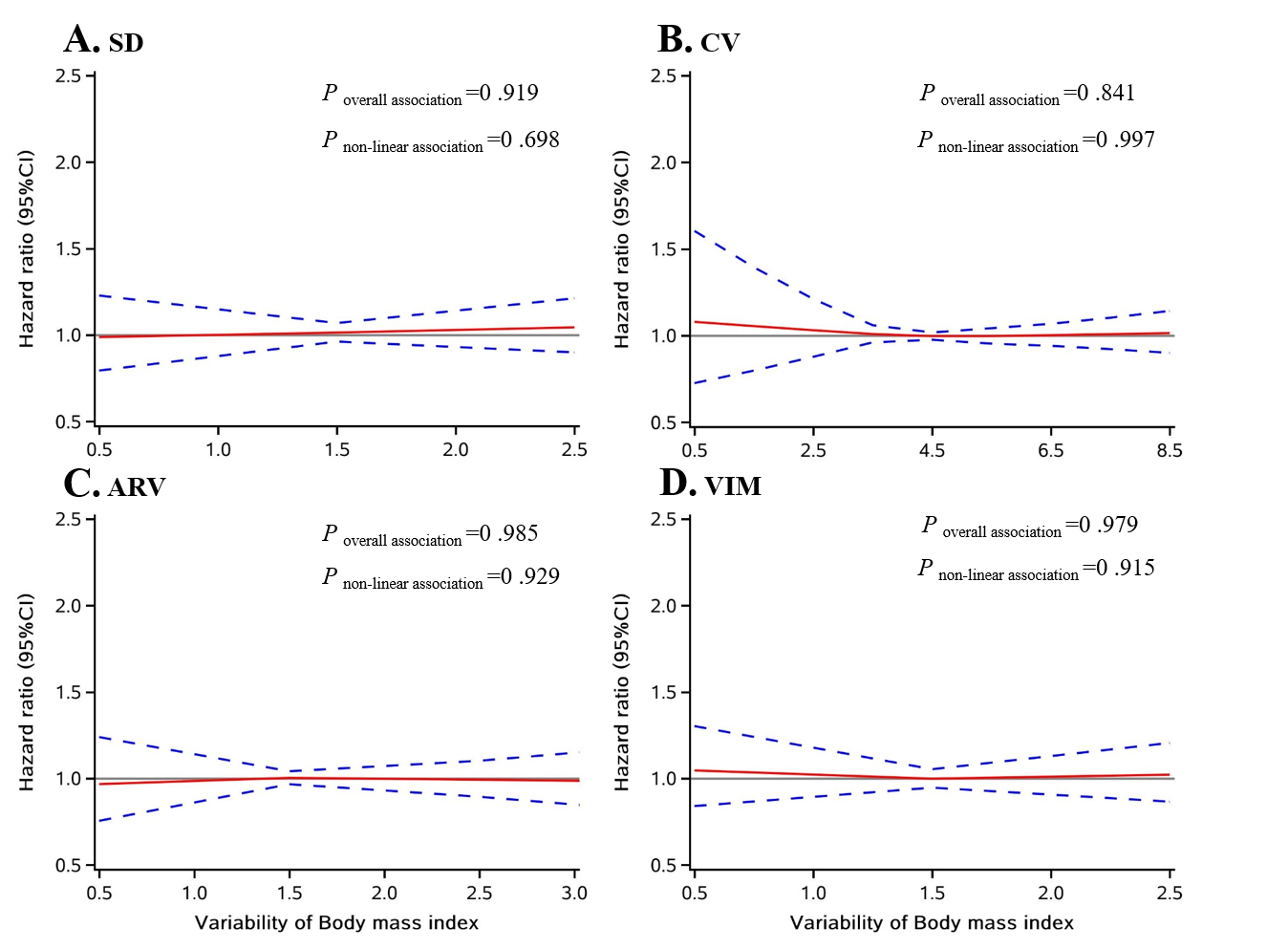


**Supplemental Fig. S2.** **Associations of BMI variability with risk of atrial fibrillation using restricted cubic spline regression models.** Point estimates (solid line) and 95% confidence intervals (dashed lines) were obtained by restricted cubic spline models with knots at the 25th, 50th, and 75th percentiles. All models were adjusted for age, sex, physical activity, smoking status, alcohol drinking status, low-density lipoprotein cholesterol, heart rate, hypertension, and diabetes. ARV, average real variability; BMI, body mass index; CV, coefficient of variation; CI, confidence interval; HR, hazard ratio; SD, standard deviation; VIM, variability independent of the mean.


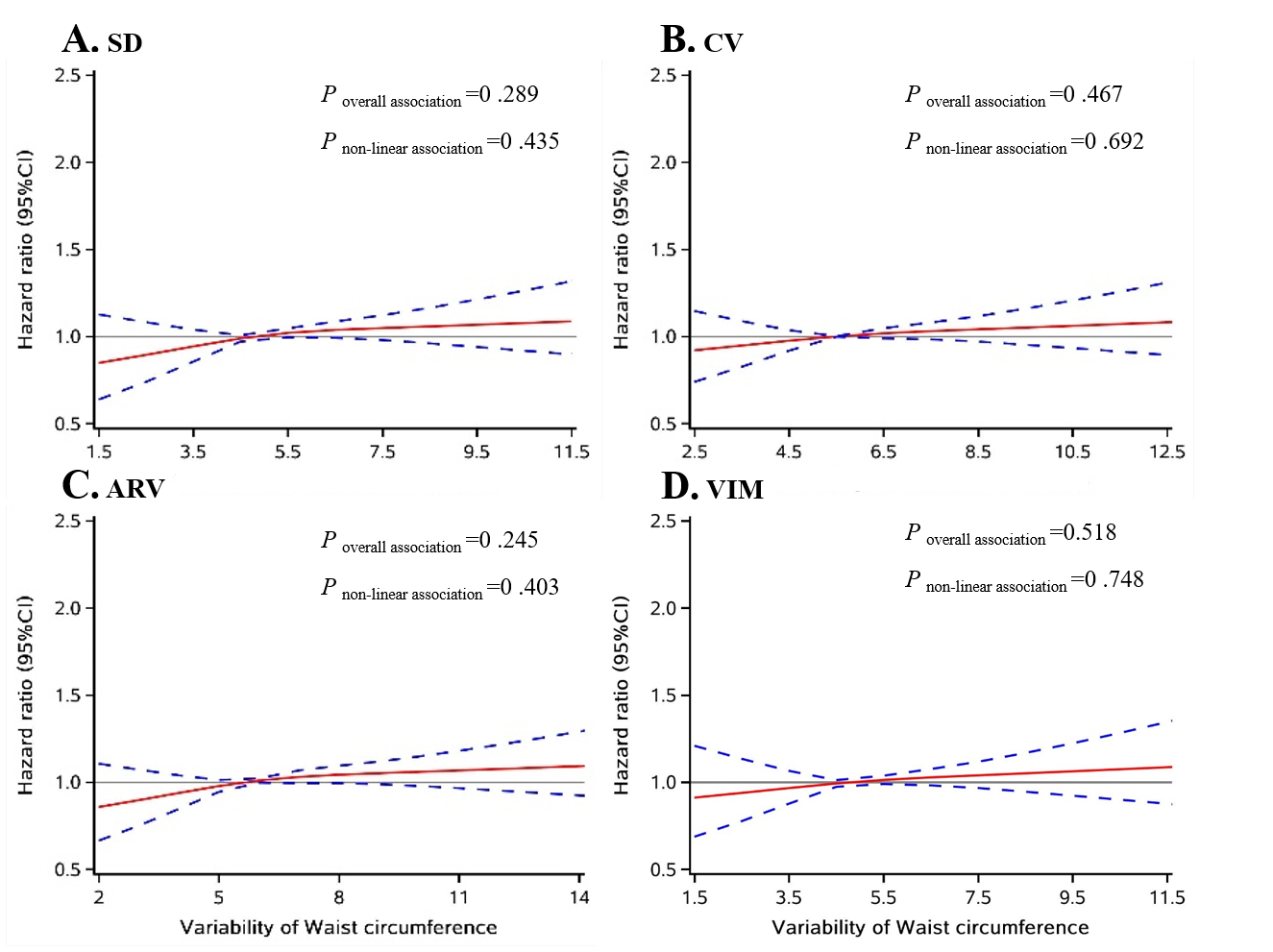


**Supplemental Fig. S3.** **Associations of WC variability with risk of atrial fibrillation using restricted cubic spline regression models.** Point estimates (solid line) and 95% confidence intervals (dashed lines) were obtained by restricted cubic spline models with knots at the 25th, 50th, and 75th percentiles. All models were adjusted for age, sex, physical activity, smoking status, alcohol drinking status, low-density lipoprotein cholesterol, heart rate, hypertension, and diabetes. ARV, average real variability; CV, coefficient of variation; CI, confidence interval; HR, hazard ratio; SD, standard deviation; VIM, variability independent of the mean; WC, [waist circumference](C:/Users/zmx/Documents/%E8%BD%AF%E4%BB%B6/Dict/8.9.9.0/resultui/html/index.html#/javascript:;).
